# Cluster‐randomized controlled trials: A tutorial

**DOI:** 10.1002/cesm.12024

**Published:** 2023-09-05

**Authors:** Marty Chaplin, Kerry Dwan

**Affiliations:** ^1^ Cochrane Infectious Diseases Group Liverpool School of Tropical Medicine Liverpool UK

## Abstract

This tutorial focuses on cluster‐randomised controlled trials. We will explain what cluster‐RCTs are, why they might be used, and how to include data from cluster‐randomised controlled trials in systematic reviews. Accompanying the tutorial is a micro‐learning module, which will give you the chance to practice adjusting data from cluster‐randomised controlled trials.

Cluster randomised trials micro learning module

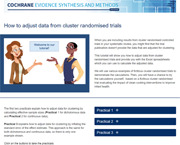

This tutorial focuses on cluster‐randomized controlled trials (cluster‐RCTs). We will explain what cluster‐RCTs are, why they might be used, and how to include data from cluster‐RCTs in systematic reviews.

**What is a cluster‐randomized controlled trial?**

In most RCTs, individuals are randomly assigned to intervention groups. In a cluster‐RCT, groups of individuals (e.g., schools, communities, or clinics) are randomized to intervention groups.

**Why use a cluster‐randomized controlled trial design?**

Table 1 outlines reasons that a cluster‐RCT design might be used by researchers, and provides an example for each of these reasons.

**Table 1:** Possible reasons for using a cluster‐randomized controlled trial design.

Reason for using cluster‐RCT design	Example
To evaluate the group effect of an intervention	To evaluate the overall effectiveness of the influenza vaccine in a population containing both people who have and have not had the vaccine (i.e. direct and indirect effects of the vaccine), groups of individuals, such as schools, could be randomized to the intervention groups.
To avoid contamination	To evaluate the effectiveness of a dietary intervention, a cluster‐RCT design may be chosen to reduce the likelihood of individuals in the control group learning about the experimental intervention, and choosing to adopt this intervention themselves. Randomizing families rather than individuals would help to reduce the risk of contamination.
Logistical reasons	To evaluate the effectiveness of insecticidal spraying on malaria prevalence, it is convenient to randomize households rather than individuals to ‘spraying’ or ‘no spraying’ as the whole household is usually sprayed
Multiple outcome measurements made on the same individual	To evaluate the effectiveness of a topical cream for a skin condition, one measurement could be taken on each arm of the same individual. Here, individuals would be randomized to interventions and each individual would be considered to be a cluster.

**How do I perform risk of bias assessments for cluster‐randomized controlled trials?**

An adaptation of the Risk of Bias 2 tool [1] outlines issues that should be considered when assessing the risk of bias of cluster‐RCTs. Detailed guidance on the use of the adapted tool is also available [2].

**What is a “unit‐of analysis” error?**

Individuals from the same cluster are likely to respond in a similar way to each other, and therefore observations made for these individuals cannot be assumed to be independent. It is important that this dependency is accounted for when analyzing data from a cluster‐RCT.

If the effects of clustering are ignored, and the analysis is conducted as if individuals were randomized, a “unit‐of‐analysis error” occurs [3], as the unit of analysis (the individual) is different to the unit of randomization (the cluster). When a “unit‐of‐analysis error” occurs, confidence intervals for the effect estimate will be artificially narrow and associated *p*‐values will be artificially small. The trial will also have too much weight in any meta‐analysis. Incorrect conclusions may therefore be drawn from the results of the cluster‐RCT itself, and any meta‐analyses that include the cluster‐RCT.

**How do I include data from a cluster‐randomized controlled trial in a systematic review?**

When a cluster‐RCT is included in a systematic review (with or without a meta‐analysis), it is important that the effect estimate and its corresponding confidence interval are adjusted for the clustered nature of the data.

The ideal approach is to extract cluster‐adjusted effect estimates and a measure of uncertainty (i.e., confidence interval or standard error) that have been calculated by the trial authors using statistical methods such as multilevel models or generalized estimating equations. These effect estimates and the measure of uncertainty may be included in meta‐analyses that use the generic inverse variance method.

Another acceptable approach is to conduct the analysis at the cluster level. The data set for analysis would include a summary measurement for each cluster, and the sample size is the number of clusters. These data can then be treated as if they were from an RCT that randomized individuals (individual‐RCT); the standard formulae can be used to obtain effect estimates and confidence intervals, and if appropriate, the data can also be included in meta‐analysis. A limitation of this approach is that sample size (and consequently, precision, and power) for the cluster‐RCT may be greatly reduced.

If it is not possible to obtain estimates and confidence intervals/standard errors that account for clustering from the trial publication or from contact with the trial authors, there are two approaches that review authors can use to obtain results that are approximately adjusted for clustering. These approaches can be used for dichotomous or continuous outcomes:

1) Calculate effective sample sizes.

This approach involves calculating the effective sample size for each arm of the trial. The effective sample size can be defined as the sample size that would be required for an individual‐RCT to have the same power and precision as the cluster‐RCT [4].

The effective sample size for each arm of the trial can be calculated by dividing the original sample size by the design effect:

Design effect=1+(M−1)ICC,

where M is the average cluster size in the trial and ICC is the intracluster correlation coefficient.

The ICC is a measure of the similarity of individuals within the same cluster with regard to a particular outcome [5], and is typically small. The ICC may be reported in the trial publication, or may be obtained from contact with trial authors. The design effect is usually the same for both arms of the trial.

For dichotomous outcomes, the number of individuals experiencing the event in each arm of the trial should also be divided by the same design effect. For continuous outcomes, means and standard deviations should remain unchanged.

Once these adjustments have been made, the data can be treated as if they were from an individual‐RCT; the standard formulae can be used to obtain effect estimates and confidence intervals, and if appropriate, the data can also be included in meta‐analysis.

2) Inflate the standard error of the effect estimate.

If review authors have an effect estimate and standard error that are not adjusted for clustering, the standard error can be multiplied by the square root of the design effect (as defined above) to obtain a standard error that accounts for the clustering effect. Standard errors can be calculated from confidence intervals, and vice versa, as described in Chapter 6.3 of the Cochrane Handbook [6]. The effect estimate and adjusted standard error or confidence interval may then be included in meta‐analyzes that use the generic inverse variance method.

**Common queries**

*What if the ICC is not reported?*

If the ICC is not available in the trial publication or from contact with trial authors, it may be possible to borrow an ICC from a similar trial. If the ICC is borrowed or estimated, sensitivity analyses can be undertaken to investigate the impact on analysis results of varying the ICC within plausible limits.

*What if it is not possible to obtain a cluster‐adjusted effect estimate?*

If it is not possible to obtain cluster‐adjusted effect estimates using any of these methods, and the review authors wish to present an unadjusted effect estimate in the text, tables, or meta‐analyses, it is important to highlight that the confidence interval for the effect estimate is likely to be too narrow due to the lack of cluster‐adjustment. If review authors do include unadjusted effect estimates from cluster‐RCTs in meta‐analyzes, sensitivity analyses should be performed to explore the impact of excluding these unadjusted effect estimates from the meta‐analysis. It is also perfectly reasonable to exclude unadjusted effect estimates from meta‐analyses completely.

*Can I include cluster‐RCTs and individual‐RCTs in the same meta‐analysis?*

Theoretically, yes. It may be informative to perform stratified or subgroup analyses by unit of randomization (I.e. cluster or individual) to investigate whether the intervention effect varies between individual‐RCTs and cluster‐RCTs. For example, in a vaccine trial, the vaccine may be more effective if administered to all individuals within a village, rather than to only some individuals.

**Further reading and online content**

More information on cluster‐RCTs, can be found in Chapter 23.1 of The Cochrane Handbook for Systematic Reviews of Interventions [6].

Cochrane Training have produced a micro‐learning module on how to adjust data from cluster‐RCTs to accompany this article [7] (Figure 1).

Professor Bland and Dr Kerry discuss cluster‐randomized controlled trials in more detail in various papers published in the BMJ [8, 9, 10, 11].

**Figure 1:**
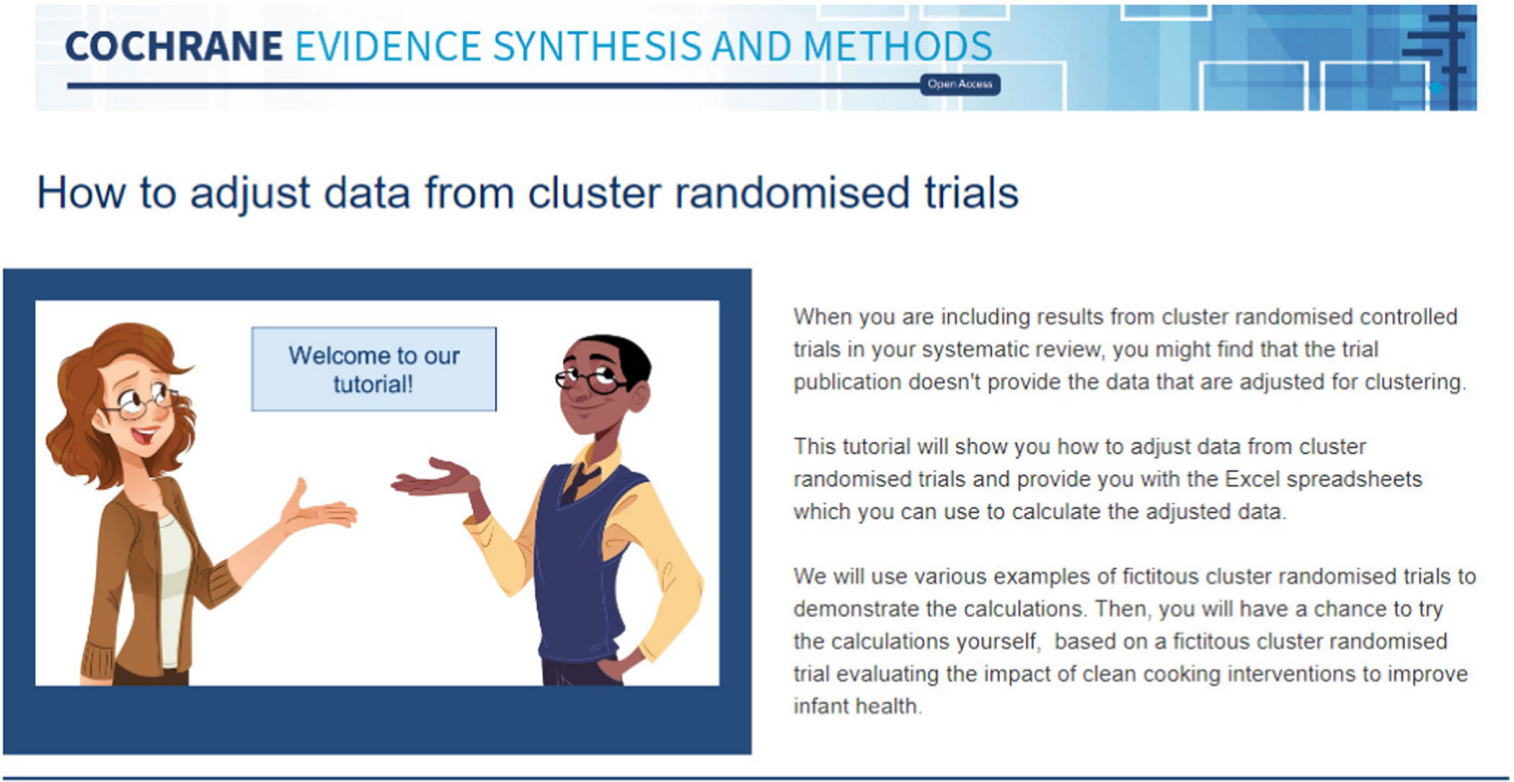
Screenshot of micro‐learning module.

## AUTHOR CONTRIBUTIONS

**Marty Chaplin**: Conceptualization; writing—original draft; writing—review and editing. **Kerry Dwan**: Conceptualization; supervision; writing—review and editing.

## CONFLICT OF INTEREST STATEMENT

The authors declare no conflict of interest.

## PEER REVIEW

The peer review history for this article is available at https://www.webofscience.com/api/gateway/wos/peer-review/10.1002/cesm.12024

## Data Availability

Data sharing is not applicable—no new data generated.
